# Anti‐skin aging effect of sea buckthorn proanthocyanidins in D‐galactose‐induced aging mice

**DOI:** 10.1002/fsn3.3823

**Published:** 2023-12-07

**Authors:** Xinying Liu, Michael Yuen, Tina Yuen, Hywel Yuen, Min Wang, Qiang Peng

**Affiliations:** ^1^ College of Food Science and Engineering Northwest A&F University Yangling China; ^2^ Puredia Limited Xining China

**Keywords:** anti‐skin aging, D‐galactose, oxidative stress, sea buckthorn proanthocyanidins

## Abstract

Oxidative stress in skin cells caused by changes in the external environment is one of the principal causes of skin aging. Sea buckthorn proanthocyanidins (SBPs) have good free radical scavenging ability. We established a senescence model by injecting 500 mg/kg D‐galactose into the dorsal necks of mice, and then different doses of SBP (25, 50, and 100 mg/kg) were gavaged to explore the effects of SBP on the skin tissues of senescent mice and elucidate the related mechanism of action. The results reveal that SBP can alleviate the skin aging phenomenon caused by D‐galactose‐induced aging. It can also enhance the total antioxidant capacity in the body, thereby strengthening the body's antioxidant defense capability. In addition, SBP can effectively improve skin aging by regulating the TGF‐β1/Smads pathway and MMPs/TIMP system, increasing the relative content of Col I and tropoelastin, further maintaining the stability of collagen fiber and elastic fiber structure. These results will provide the development and production of the antioxidant function of cosmetics and health products, providing a new train of thought.

## Graphical abstract

This paper provides an in‐depth analysis of the potential role of sea buckthorn procyanidins in delaying skin aging. The core mechanism lies in reducing oxidative stress in the body, effectively maintaining the relative content of type I collagen and tropoelastin, while maintaining the stability of the skin’s fiber structure. Additionally, sea buckthorn procyanidins can also increase the skin’s moisture content and hyaluronic acid content, further delaying skin aging.

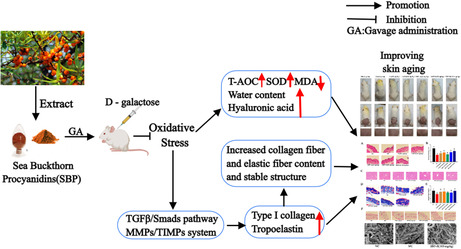

## 1 INTRODUCTION

With the development of society, more and more attention is being paid to the issue of how to maintain skin health and delay skin aging. Skin plays a very significant role in resisting the influence of the external environment and maintaining homeostasis (Franco et al., 2022). Its aging is an unavoidable gradual process, regardless of the causes of skin aging; dull skin, thinning, wrinkle production, and reduced elasticity are typical of skin aging, which is mainly determined by the relative content of water and hyaluronic acid, collagen, and elastin in the extracellular matrix (ECM) of the dermis and the structural stability of collagen fibers and elastin fibers (Lee et al., 2008). Aging models established by administering high doses of D‐galactose stimulation to rodents have been confirmed by domestic and international academics. They are widely used in the field of medical anti‐aging research (Rusu et al., 2020). It can disturb the redox homeostasis balance of the body, thus damaging the homeostasis of the skin, resulting in the loss of skin water content, collagen, and elastin, and ultimately causing the occurrence of skin aging (Azman & Zakaria, 2019; Umbayev et al., 2020; Zhang et al., 2020).

Sea buckthorn (*Hippophae, Elaeagnacea*) is widely distributed in Eurasia (Ciesarová et al., 2020). Due to its rich nutritional value and medicinal properties, it has gained significant attention in the pharmaceutical and food industries. They have been found to protect retinal cells (Wang et al., 2021), prevent or treat diabetic nephropathy (Gong et al., 2022), and maintain cardiovascular health by lowering blood pressure, inhibiting platelet aggregation, and reducing the risk of atherosclerosis (Du et al., 2021), among other benefits. In addition, proanthocyanidins have shown positive effects on skin health. Due to their excellent antioxidant properties, they help reduce skin damage and inflammation and reduce wrinkles and sagging skin signs of aging, resulting in a more youthful and elastic appearance (Cocetta et al., 2021). In our previous studies, we completed the isolation and identification of Sea buckthorn proanthocyanidins (SBP) and found that it is a source of low‐molecular‐weight proanthocyanidins with good free radical scavenging ability (Zhu et al., 2021). In order to investigate the possible application of SBP in skin aging, we pre‐investigated and found through cellular experiments that SBP could exert the anti‐cellular senescence effect by increasing the relative content of type I collagen (Col I) in H_2_O_2_‐induced senescent cells of human dermal fibroblasts (Liu et al., 2022). To further examine its feasibility in preventing skin aging and further increase the market application value of SBP, we established a senescence model by subcutaneously injecting D‐galactose into mice and then gavage with different doses of SBP to explore the effects of SBP on the skin tissues of senescent mice and to elucidate the related mechanism of action.

## 2 MATERIALS AND METHODS

### 2.1 Chemicals and reagents

Sea Buckthorn Proanthocyanidins (SBP, Purity: 91.5%, Trademark: CyanthOx™) were supplied from Puredia Limited (Qinghai, China). Grape Seed Proanthocyanidins Extract (GSPE) was purchased from Merck Group (Darmstadt, Germany, cat. no. 1298208). Primary antibodies: anti‐Smad 3 was purchased from Proma (Hunan, China, cat. no. 30072); anti‐TGF‐β1 was purchased from ABclonal (Wuhan, China, cat. no. A2124); and anti‐type I collagen (cat. no. ab260043), anti‐tropoelastin (cat. no. Ab217356), anti‐LOX (cat. no. ab174316), and anti‐Fibulin 5 (cat. no. ab109428) were purchased from Abcam (Cambridge, MA, USA). β‐Actin was purchased from Protein Group, Inc. (Wuhan, China, cat. no. 66009‐1‐Ig). Secondary antibodies: rabbit IgG (H + L) antibody (peroxidase‐labeled anti‐mouse) (cat. no. 074–1506) and IgA + IgG + IgM (H + L) antibody (human serum adsorbed and peroxidase‐labeled) (cat. no. 074–1807) were purchased from KPL (Maryland, Washington, UK). The test kit: superoxide dismutase (SOD, cat. no. S0101S), total antioxidant capacity (T‐AOC, cat. no. S0119), and malondialdehyde (MDA, cat. no. S0131S), were all purchased from Beyotime (Shanghai, China). Enzyme‐linked immunosorbent assay (ELISA) assay kits: Matrix Metalloproteinase‐1 (MMP‐1, cat. no. E‐EL‐M0779c), Matrix Metalloproteinase‐3 (MMP‐3, cat. no. E‐EL‐M0626c), Matrix Metalloproteinase‐9 (MMP‐9, cat. no. E‐EL‐M3052), and Tissue Inhibitor of Metalloproteinase‐1 (TIMP‐1, cat. no. E‐EL‐M0641c) were purchased from Elabscience Biotechnology Co., Ltd. (Wuhan, China). D‐galactose (cat. no. SG8010), hyaluronic acid (HA) kits (cat. no. G3710), and Vitamin C (Vc, cat. no. A8100) were purchased from Bei Jing Solarbio Science & Technology Co., Ltd. (Beijing, China). The remaining reagents are of analytical grade.

### 2.2 Animals and treatment

**FIGURE 1:**
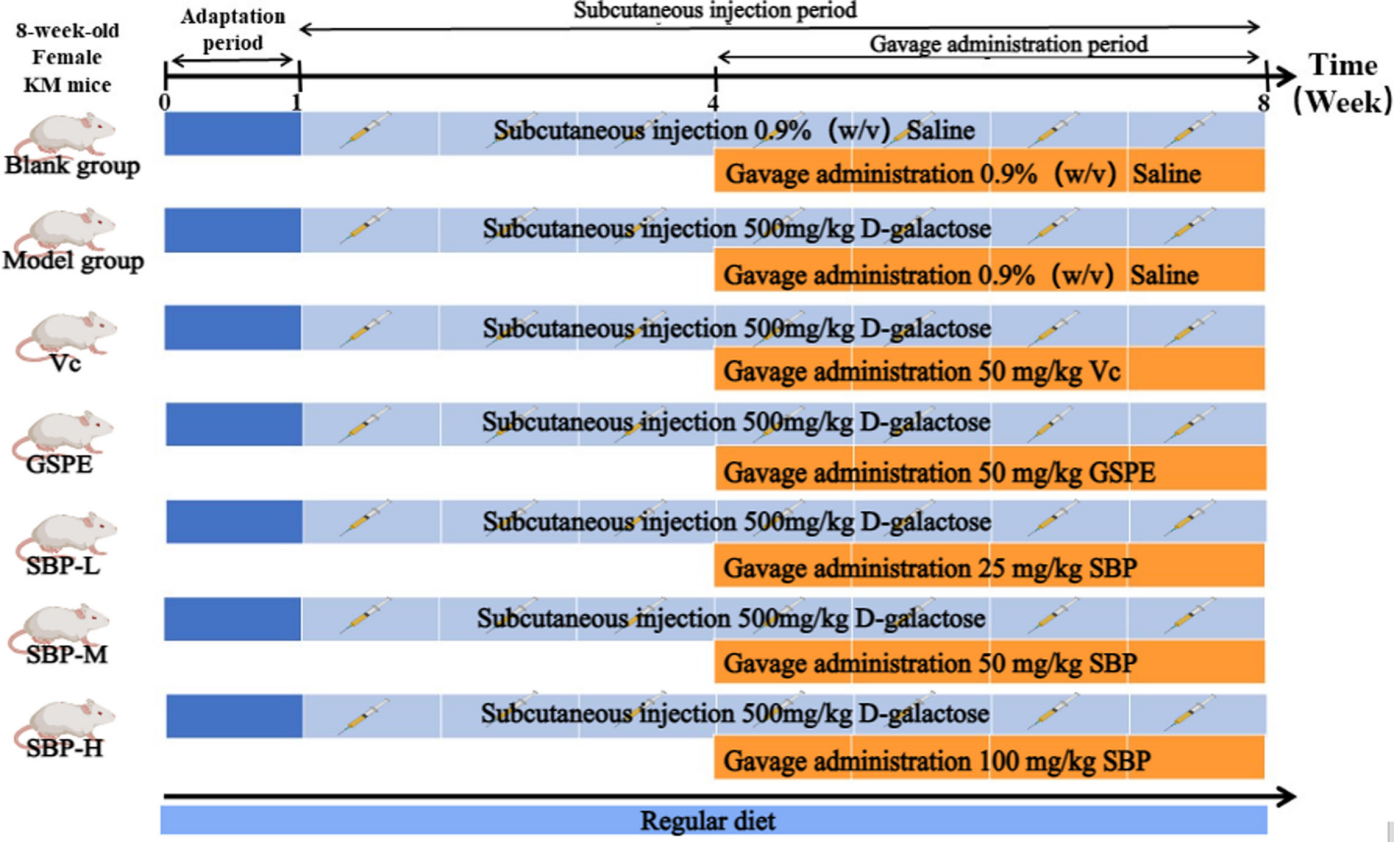
Illustrative diagrams of animal experimental procedures.

Eight‐week‐old female Kunming mice, weighing approximately 30 ± 2 g, were received from the Animal Centre of Xi'an Jiaotong University (Shaanxi, China). In a standard laboratory with 60% (relative humidity) and 25 ± 2°C, mice are alternated between 12 h light and 12 h dark treatments and can freely obtain water. We then randomly divided the mice into seven groups of eight per group after 7 days of adaptation feeding. Throughout the experiment, a record of the body weight and skin thickness (measured with an electronic vernier caliper after removing the dorsal hair) of each mouse every weekday was maintained. Except for the normal control (NC) group, 0.3 mL of normal saline was injected subcutaneously. The other groups were injected with the same volume of D‐galactose (500 mg/kg) once a day for 7 weeks. Starting from the fourth week, except for the NC group, which was gavaged with the same volume of distilled water as the aging model control (MC) group every day, the other five groups were gavaged 25, 50, and 100 mg/kg of SBP (SBP‐L, SBP‐M, SBP‐H), 50 mg/kg of Vc, and 50 mg/kg of GSPE every day, respectively. The experiment was performed as shown in Figure 1. At the designated experimental endpoints, mice were anesthetized, and their dorsal hair was removed, followed by cervical dislocation. Immediately after the mice were sacrificed, whole blood, dorsal skin, and some liver tissues were collected, and some of the organs were weighed for subsequent experiments.

Female mice were selected for this experiment because the attention of female mice to bullying aging was slightly greater than that of male mice. The whole experiment was conducted in accordance with all relevant ethical codes in animal experiments and was reviewed and approved by the Animal Policy and Welfare Committee of Northwest A&F University (Yangling, Shaanxi, China, approval no. XN2023‐0603).

### 2.3 Skin aging score

Throughout the study period, three independent researchers, once a week on Sunday, scored each rat for aging after partial de‐hairing of the back, as shown in Table 1 (Chawalitpong et al., 2019; Yoshimura et al., 2021).

**TABLE 1:** Skin aging scoring criteria.

Score	Skin aging scoring criteria
1–10	No folds or sagging, no thickening, longitudinal streaks running down the back and appearing or disappearing with movement; skin is free of pigmentation, shiny and soft; no hair loss, hair is abundant and shiny
11–20	Most of the skin has fine longitudinal stripes, and a few transverse fine stripes can appear or disappear with movement; a small amount of pigmentation exists in the skin, the degree of brightness and tenderness is slightly worse, the phenomenon of hair loss is present but not evident, the degree of vigorous and oily hair is worse
21–30	All fine longitudinal lines disappear; a small number of transverse shallow wrinkles appear, do not appear or disappear with movement; a large number of skin pigmentation, rougher, hair loss phenomenon aggravated, hair brightness significantly reduced
31–40	Persistence of a large number of horizontal roughness and deep wrinkles and noticeable sagging of the skin; severe skin pigmentation, apparent roughness, and dullness without shine; profound hair loss and no shine

### 2.4 Measurement of skin water content

After the mice were sacrificed, 1 cm^2^ of skin was cut as much as possible, and its wet weight was weighed precisely. Then, it was put into the oven to dry (80°C, 12 h) and weighed, and then the skin moisture percentage was calculated using the following formula:

$$\text{Skin water content percentage}=\left(\mathrm{Wet}\;\text{weight}-\mathrm{Dry}\;\text{weight}\right)/\mathrm{Wet}\;\text{weight}\times 100\%$$

### 2.5 H&E staining, Masson staining, Victoria blue staining of skin tissue, H&E staining of liver tissue, and pathological damage score

After sacrificing the mice, the skin and a portion of the liver tissue were collected. They were fixed for 48 h (by using 4% paraformaldehyde) and then for paraffin embedding operations subsequently. Next, a microtome was used to prepare thin sections with a thickness of 4–6 μm. Three kinds of pathological staining were performed on the skin tissue sections: H&E staining, Masson's staining, and Victoria blue staining.

The liver tissue sections were subjected to H&E staining. After dehydration and mounting, the sections were observed using an inverted microscope, and the pathological scores of liver tissue injuries were evaluated. Five fields of view were taken from each section, and scores were assigned based on the degree of congestion, the number of polymorphonuclear leukocytes, and the presence of cell death (nuclear fragmentation, nuclear lysis, nuclear condensation, and vacuolar degeneration) within the field of view. The scores were based on the above phenomena and ranged from 0 to 5 points [0, none; 1, rare (<1% of total cells); 2, occasional (1%–10% of scattered points); 3, regular (1%–10% of the entire section); 4, common (10%–50% of the entire section); and 5, very common (>50%)] (Kleiner et al., 2005; Zhang et al., 2023).

### 2.6 Measurement of organ coefficients in mice

Once the mice have been sacrificed, the heart, liver, spleen, lungs, and kidneys are extracted. These organs are rinsed using a cold saline solution and subsequently dried using filter paper. The next step involves weighing the organs for the purpose of organ coefficient analysis.

$$\text{Organ coefficient}=\left(\text{Organ weight}/\text{Body weight}\right)\times 100$$

### 2.7 Measurement of oxidative stress levels

Upon sacrificing the mice, whole blood is promptly collected and subjected to centrifugation at 2200 xg (4°C, 10 min) to isolate the serum. The levels of T‐AOC, SOD, and MDA in the serum and skin tissue are measured using specific assay kits, following the manufacturer's recommended protocols. All experimental procedures adhere to the guidelines provided by the manufacturer.

### 2.8 Measurement of HA, MMP‐1, MMP‐3, MMP‐9, TIMP‐1 content

ELISA is a highly sensitive and specific technique used for quantitative analysis of target molecules. It was employed to measure the levels of HA, MMP‐1, MMP‐3, MMP‐9, and TIMP‐1 in skin tissue.

### 2.9 Scanning electron microscopy analysis

According to the method of Chu et al. (2022)), with slight modifications, mice skin tissues were cut to 2 × 2 cm^2^ and fixed with 2.5% glutaraldehyde for more than 5 h, then washed with 0.1 M PBS buffer pH 6.8, soaked four times for 10 min each, with a dosage of about 2 mL each, followed by gradient dehydration with 30%, 50%, 70%, 80%, 90%, and 100% ethanol solutions twice each for 10 min. CO_2_ drying was then performed. After drying, the dried samples were fixed on the sample plate after gold spraying treatment, and then the microstructure was observed using a scanning electron microscope (Nano SEM‐450).

### 2.10 Western blotting

The skin tissue was disrupted into homogenates using RIPA lysis buffer (Beyotime, Shanghai, China, cat. no. P0013B). Following the method described by Liu et al., 2022, the BCA protein assay kit (Beyotime, Shanghai, China, cat. no. P0012S) was utilized for protein quantification. The same amount of protein standard solution was loaded into the corresponding swim lanes, and primary antibody incubation was performed after membrane transfer. The primary antibodies used included anti‐TGF‐β1 (1:2000), anti‐type I collagen (1:1000), anti‐Smad3 (1:2000), anti‐tropoelastin (1:2000), anti‐LOX (1:2000), anti‐Fibulin 5 (1:2000), and anti‐β‐actin (1:5000). Subsequently, a 1‐hour incubation with secondary antibodies was carried out at room temperature. The visualization of protein bands was accomplished using the ECL protein immunoblot detection kit (1:5000, Beyotime, Shanghai, China, cat. no. P0018AS), and quantification was performed utilizing Image J software.

### 2.11 Statistical analysis

All data were analyzed using GraphPad Prism Version 8 (GraphPad Software, San Diego, US) and IBMSPSS Statistics 22.0 (SPSS Inc., Chicago, IL, US) software and expressed as mean ± standard value (SD). The differences between the data sets were compared using an ANOVA and a T‐test.

## 3 RESULTS

### 3.1 The effects of SBP on body weight, skin thickness, and skin aging score in aging mice

**FIGURE 2:**
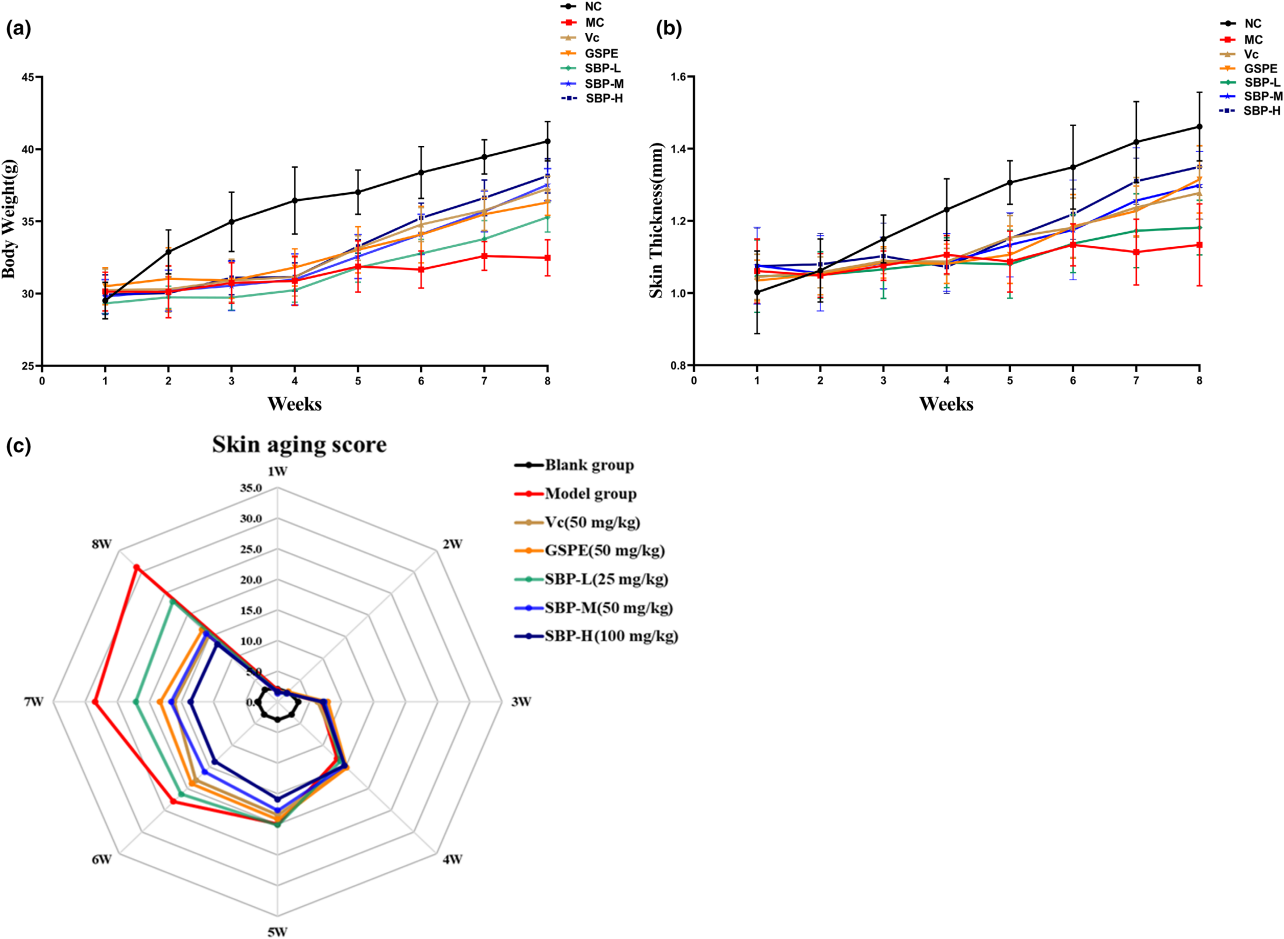
The effect of SBP on body weight, skin thickness, and skin aging score in aging mice. (a) The effect of SBP on the body weight of aging mice. (b) The effect of SBP on the skin thickness of aging mice. (c) The effect of SBP on skin aging scores.

During the aging modeling period, the change in mice's body weight gain is a critical indicator of their health status, growth, and development. The data in Figure 2a indicates that long‐term injection of D‐galactose (500 mg/kg) adversely affected the growth and development of mice and that higher concentrations of SBP (50 and 100 mg/kg) could reverse this adverse effect.

Skin aging can lead to skin thinning or an increase in aging scores. As shown in Figure 2b, the difference in skin thickness between the blank group and the D‐galactose‐injected group of mice was gradually evident during the first 3 weeks of the test cycle, with a gradual increase in skin thickness in the blank group and no significant difference between the remaining D‐galactose‐injected groups. According to the skin aging scoring standard in Table 1, the aging condition of the back skin of the mice was scored at the end of every week. Compared with the NC group, the back skin of mice in the MC group showed fine lines, pigmentation, stiffness, lack of elasticity, and other symptoms similar to aging, and increased skin aging score, which indicated that the skin aging model had been established successfully. Through the treatment of Vc, GSPE, and SBP drugs, the wrinkles on the back of mice became lighter, and the elasticity and glossiness increased significantly, decreasing the skin aging score. According to the data shown in Figure 2c, starting from the fourth week, the skin thickness of mice in all four groups, Vc, GSPE, SBP‐M, and SBP‐H, gradually increased and recovered, and the aging score also gradually decreased. Except for the MC and SBP‐L groups, which showed a less obvious trend of increase, the increase was the most in the SBP‐H group, closest to the blank group. These results indicated that the aging of the skin was gradually alleviated with the increase in SBP dose. This may be because SBP, as an exogenous supplement of natural antioxidants, can effectively resist oxidative stress damage caused by the significant accumulation of ROS in skin cells caused by the intake of D‐galactose beyond the metabolic range of the body, thereby delaying the appearance of skin aging problems.

### 3.2 The effect of SBP on organ coefficients in mice

The change in visceral coefficient is an essential indicator of organism aging. As shown in Table 2, D‐galactose could induce organismal senescence, significantly decrease the organ coefficients of the heart, liver, spleen, and kidney, and increase the organ coefficients of the lung in the model group. All VC, GSPE, and SBP drug groups had no more significant effect on the heart, spleen, lungs, and kidneys than the blank group. However, the liver coefficients showed significant differences between the groups. This suggests that the liver is the main metabolic organ for VC, GSPE, and SBP and may exert their significant anti‐aging effects through the liver.

**TABLE 2:** The effect of SBP on organ coefficients in mice.

Experimental groups	Heart	Liver	Spleen	Lung	Kidney
NC	0.55 ± 0.10^a^	4.12 ± 0.54^a^	0.42 ± 0.12^a^	0.64 ± 0.08^b^	1.31 ± 0.10^a^
MC	0.42 ± 0.06^b^	3.28 ± 0.19^d^	0.30 ± 0.06^bc^	0.79 ± 0.11^a^	1.07 ± 0.14^b^
Vc	0.53 ± 0.06^ab^	3.87 ± 0.38^abc^	0.33 ± 0.06^ab^	0.68 ± 0.17^ab^	0.19 ± 0.16^ab^
GSPE	0.55 ± 0.14^a^	3.49 ± 0.80^bcd^	0.34 ± 0.04^ab^	0.67 ± 0.13^ab^	1.16 ± 0.17^ab^
SBP‐L	0.53 ± 0.05^ab^	3.21 ± 0.34^d^	0.24 ± 0.10^c^	0.72 ± 0.19^ab^	1.10 ± 0.15^b^
SBP‐M	0.52 ± 0.11^ab^	3.41 ± 0.40^cd^	0.32 ± 0.06^bc^	0.71 ± 0.09^ab^	1.19 ± 0.16^ab^
SBP‐H	0.59 ± 0.13^a^	3.95 ± 0.36^ab^	0.42 ± 0.11^a^	0.66 ± 0.05^ab^	1.26 ± 0.07^a^

*Note*: ^a–d^Mean values within the same row not sharing a common superscript letter are significantly different (*p* < .05).

### 3.3 The effect of SBP on antioxidant activity in skin tissue and serum of aging mice

**FIGURE 3:**
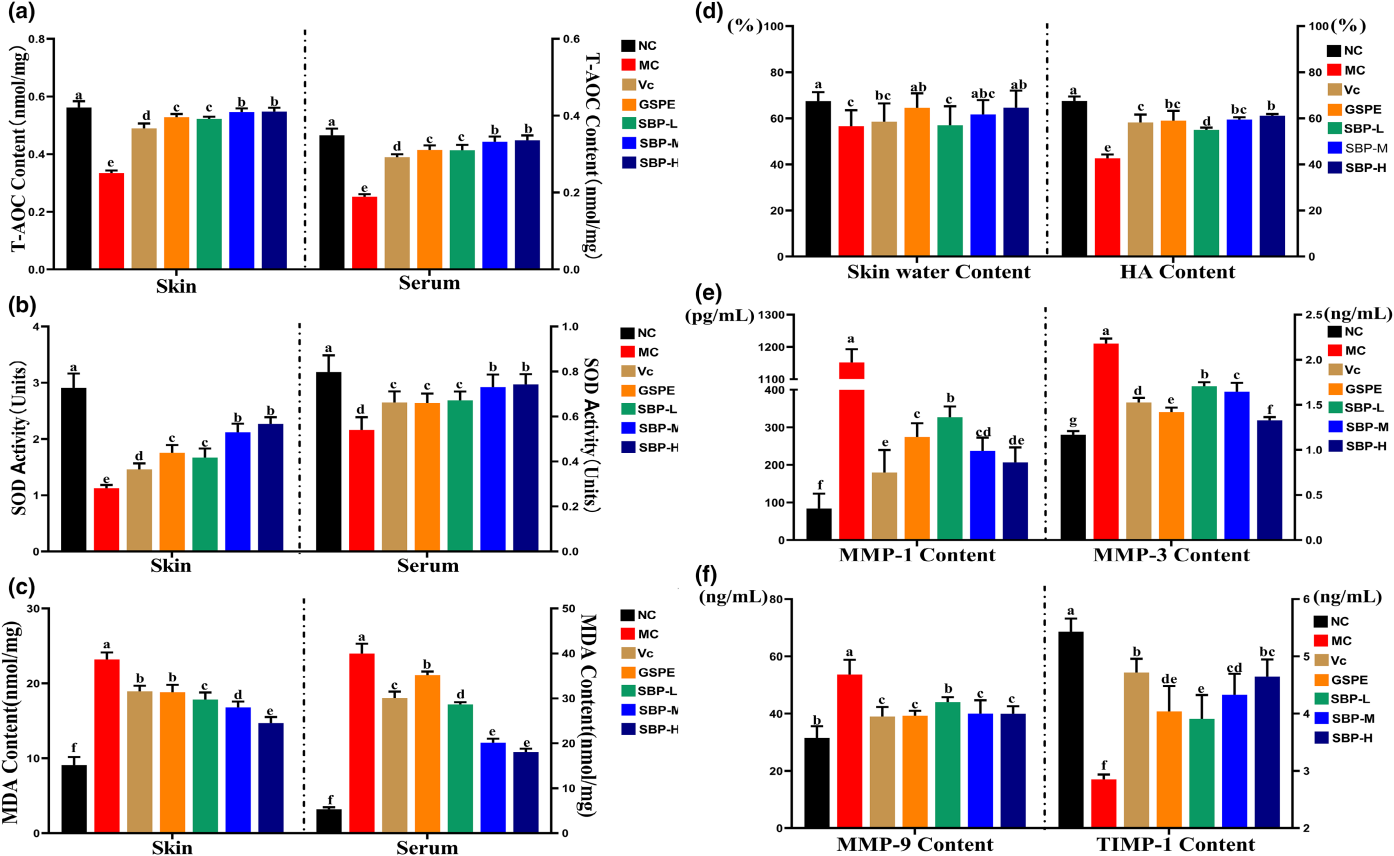
The effect of SBP on antioxidant activity in skin tissues and serum of aging mice, and the effect of SBP on the water content and HA content of skin tissues of aging mice and the degradation mechanism of Col I and tropoelastin in aging mice skin tissues. (a–c) The T‐AOC content, the activity of SOD, and the MDA content in skin tissues and serum of aging mice. (d–f) The water, HA, MMP‐1, MMP‐3, MMP‐9, and TIMP‐1 content of skin tissues of aging mice. ^a‐f^Mean values within the same row not sharing a common superscript letter are significantly different (*p* < .05) by Duncan's multiple range test.

The oxidative stress reaction in skin tissue cells due to increased ROS is connected with skin aging (Michel et al., 2012). To investigate the antioxidant effect of SBP in the skin tissues and serum of aging mice, we examined the levels of T‐AOC content, SOD activity, and MDA content in the serum and skin tissues of aging mice. In Figure 3a–c, we can observe that the T‐AOC content and SOD activity in the skin tissues and serum of the aging model decreased significantly, and the MDA content increased significantly compared with the NC group, but after the intervention of the aging model with Vc, GSPE, SBP‐L, SBP‐M, and SBP‐H groups drugs in the experimental group, as seen in figure, it is clear that the high‐dose group of SBP (50, 100 mg) significantly increased the T‐AOC levels and SOD activity compared to the aging model group, and there was no significant difference between them. The increases in the Vc, GSPE, and SBP‐L groups were slightly smaller than those in the previous two groups. While MDA content appeared to be a dose‐dependent decline in SBP, the two positive control groups, Vc and GSPE groups, were not as effective or as good as the SBP‐L group. These results indicate that SBP could enhance the capacity of antioxidant defense in mice and thus play an anti‐aging role.

### 3.4 The effect of SBP on the HA content and the water content of skin tissues in aging mice

The assessment of water content and HA levels in skin tissue is crucial for evaluating skin aging. As shown in Figure 3d, the moisture content and HA levels in the MC group were significantly lower than those in the NC group. GSPE and the high‐dose groups of SBP (50 and 100 mg) increased the water content in mice's skin, with no significant difference compared to the blank group. The HA levels in mice skin showed a similar trend to the moisture content, with the GSPE group, SBP‐M group, and SBP‐H group slightly lower than the NC group and no significant differences among these three groups. All the data results indicate that SBP and GSPE enhance the water content and HA levels in skin tissue, with better effects observed at higher doses of SBP.

### 3.5 Observation of H&E staining and epidermal layer thickness of skin tissue

**FIGURE 4:**
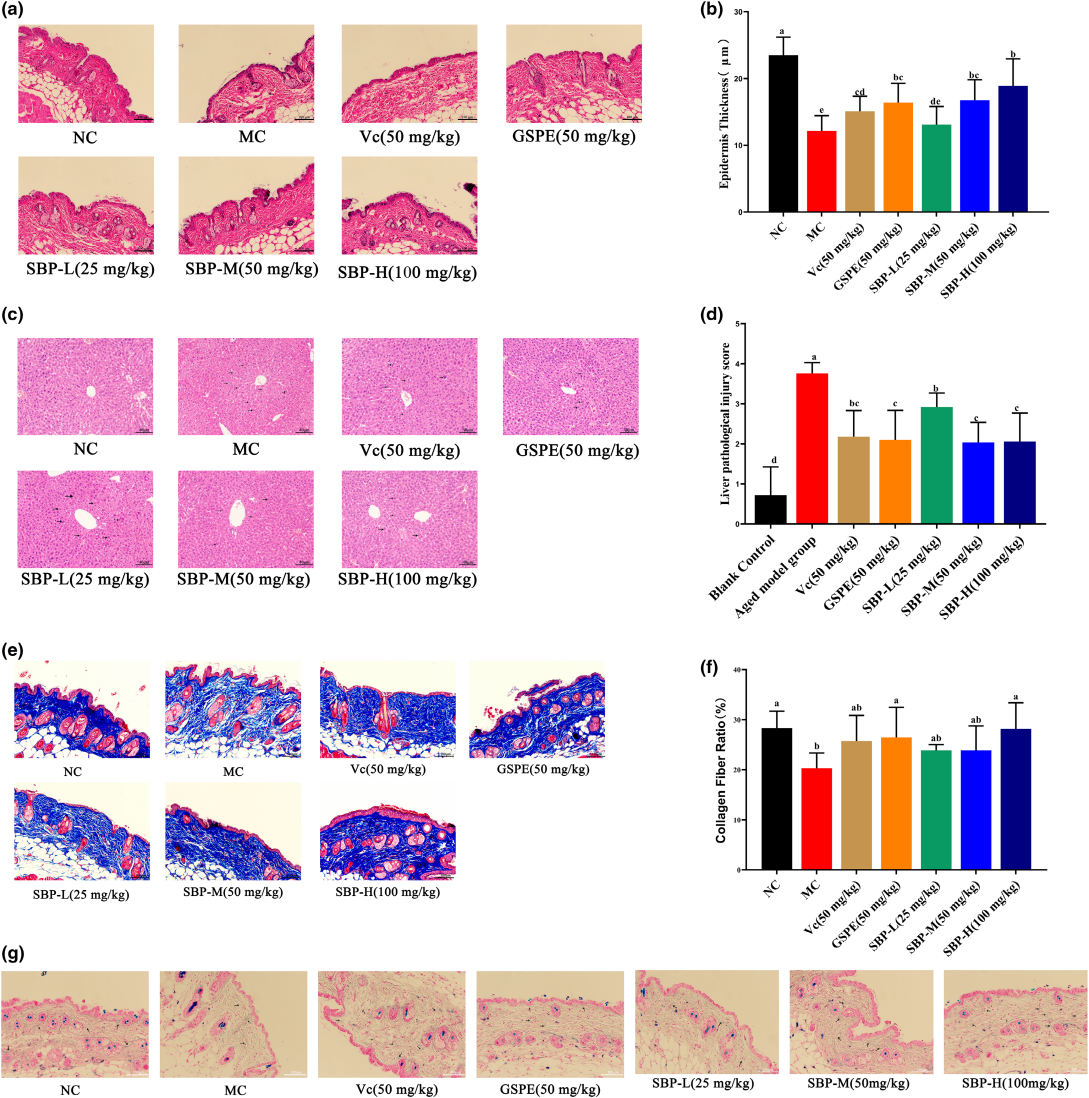
Histopathological observation of skin and liver and the effect of SBP on collagen and elastic fibers in the skin of aging mice. (a) The results of H&E staining on mice skin (200×, scale bar 100 μm). (b) Quantification of epidermal layer thickness based on skin H&E staining results. (c) The results of H&E staining on mice liver tissue (200×, scale bar 40 μm). (d) Liver pathological injury score. (e) The results of Masson staining on mice skin (200×, scale bar 100 μm). (f) Quantification of the proportion of collagen fibers based on skin Masson staining. (g) The results of Victoria blue staining on mice skin (200×, scale bar 100 μm). ^a‐f^Mean values within the same row not sharing a common superscript letter are significantly different (*p* < .05) by Duncan's multiple range test.

Figure 4a, mice skin tissue H&E staining results. The skin's epidermis in the NC group was regular, with uniform thickness, tightly arranged epidermal cells, normal morphology, and a clear boundary between the epidermis and dermis. The mice in the MC group had irregular epidermis, uneven thickness, local keratosis, and thinning of the dermis. Compared with the MC group, the skin tissue morphology of the GSPE and SBP‐M groups was improved to a certain extent, their epidermal layer morphology was more regular, and the dermal thickness was increased. In the SBP‐H group, the skin structure was intact, the epidermis was smooth, and the thickness was uniform.

We quantified the thickness of the epidermal layer of mice based on H&E staining of their skin (Figure 4b). From the figure, we can see that the GESP group, SBP‐M group, and SBP‐H group can obviously improve the effect of thinning of skin epidermal layer thickness caused by aging, which is consistent with the above results of the effect of SBP on the skin thickness of aging mice.

### 3.6 Effect of SBP on liver histopathological changes

Through analysis of the organ coefficients of mice, we found significant differences in the liver coefficients among the experimental groups. To investigate whether SBP exerts its anti‐aging effect through the liver, we performed H&E staining on liver tissue to observe the effect of SBP on pathological tissue changes and injury scores in the liver. From Figure 4c, we can know that the liver slices of the NC group mice showed no pathological changes. Compared to the NC group, the MC group mice exhibited partially indistinguishable hepatic lobule structure, disrupted hepatic sinusoidal arrangement, and loosely arranged liver cells. In contrast to the MC group, the SBP group (50 and 100 mg), Vc group, and GSPE group resulted in a more vibrant liver tissue color in mice. The hepatic lobule morphology was normal, and the liver cells near the central vein exhibited regular and tight arrangements. The hepatic sinusoids were clear, and the liver cell morphology was normal, with clear boundaries and homogeneous cytoplasm. These results are consistent with those presented by the liver injury score (Figure 4d).

### 3.7 Observation of Masson staining of skin tissue and the effect of SBP on collagen fibers and elastic fibers in the skin of aging mice

Masson staining was performed on collagen fibers in the aging mice skin, and the staining results are shown in Figure 4e; the percentage of collagen fibers is shown in Figure 4f. The collagen fibers in the dermis of the blank group were bright blue, neatly arranged in bundles, tightly ordered, and moderately thick and thin. The skin of the MC group was loosely and unevenly arranged compared with the blank group, and the number of dermal collagen fibers was reduced. In addition, some collagen was disconnected and missing. Compared with the MC group, the number of collagen fibers in the Vc, GSPE, SBP‐L, SBP‐M, and SBP‐H groups was significantly increased, with a dense and regular arrangement and corrugated shape. The overall results showed that both Vc, GSPE, and SBP increased the content of collagen fibers in skin tissues.

The results of Victoria blue staining of elastic fibers in the skin of senescent mice showed (Figure 4g) that the elastic fibers in the dermis of the blank group were light blue, more elongated, and uniformly distributed in a wavy pattern. The elastic fibers in the MC group were reduced, shorter, and structurally disordered compared with the blank group. Compared with the aging model group, the elastic fibers in the Vc and SBP‐H groups were increased and less broken, with a regular arrangement.

### 3.8 The effect of SBP on Col I and the tropoelastin synthesis pathway

**FIGURE 5:**
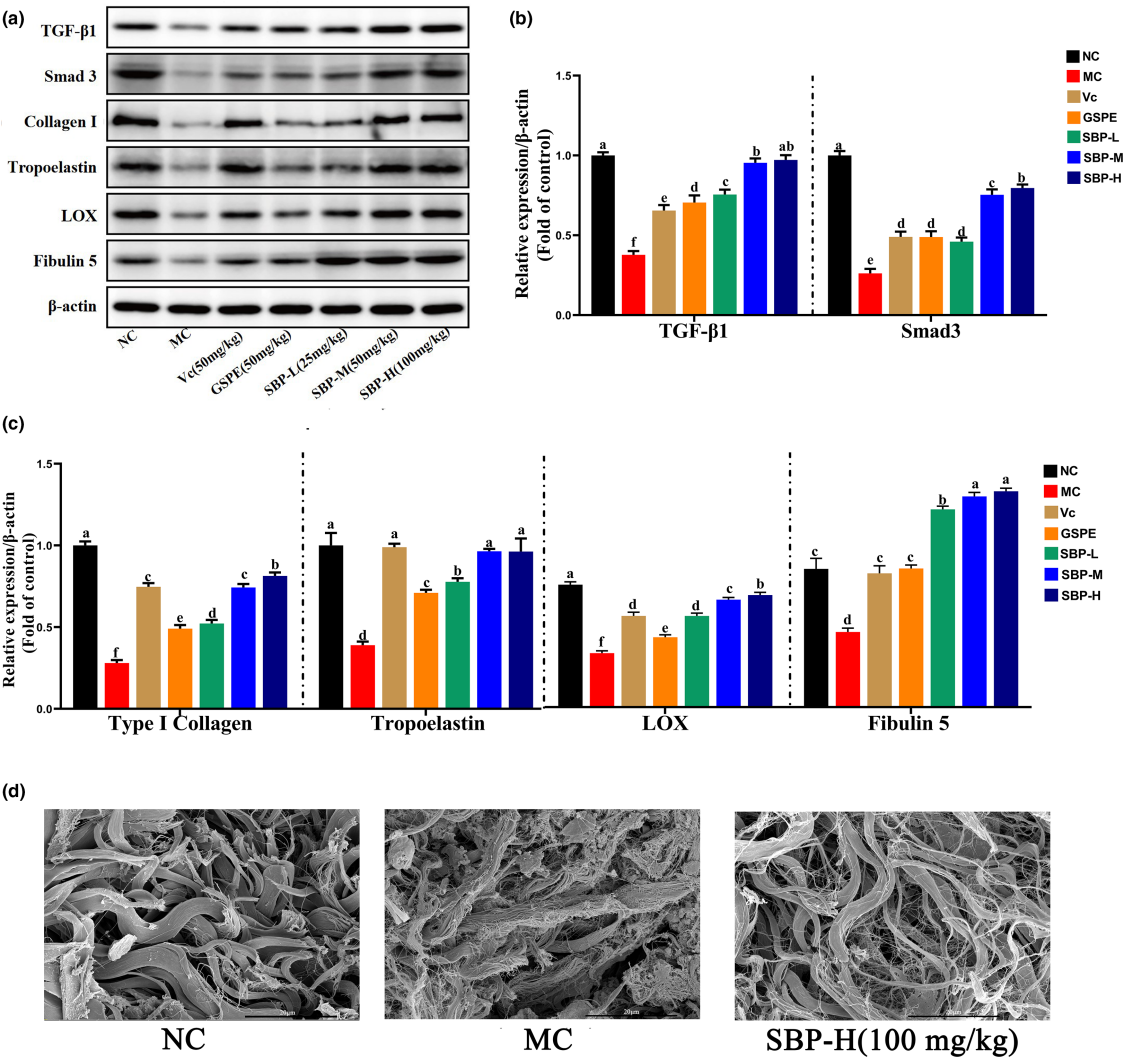
The effect of SBP on Col I and tropoelastin synthesis pathways. (a) Associated protein bands. (b, c) Expression of TGF‐β1, Smad3, Col I, Elastin, LOX, and Fibulin 5 in skin tissues of different experimental groups. Protein blots are representative of at least three independent experiments. (d) Scanning electron microscopic observation of fibrous tissue in the dermis of mice skin tissue. ^a‐f^Mean values within the same row not sharing a common superscript letter are significantly different (*p* < .05) by Duncan's multiple range test.

TGF‐β1/Smads can regulate the synthesis of Col I and tropoelastin in cells, and LOX and Fibulin 5 are indispensable in synthesizing elastic fibers (Ignotz & Massagué, 1986; Mora Huertas et al., 2016; Weihermann et al., 2017). As shown in Figure 5a–c, the levels of TGF‐β1, Smad3, Col I, tropoelastin, LOX, and Fibulin 5 in the MC group were significantly lower than those in the NC group, but, as shown in the figure, SBP reversed the effects of aging on the above proteins. The best effect was seen with medium to high concentrations of SBP. In general, SBP can promote the synthesis of Col I and tropoelastin by increasing TGF‐β1 and Smad3 in the skin of aging model mice to delay skin aging.

### 3.9 The effect of SBP on Col I and tropoelastin degradation mechanisms

In the aging process, the overexpression of MMP‐1, MMP‐3, and MMP‐9, along with the decrease in TIMP‐1, may lead to abnormal degradation of Col I and tropoelastin in the skin tissue. From Figure 3e,f, we can know that in the MC group, aging‐induced skin aging results in a significant increase in the levels of MMP‐1, MMP‐3, and MMP‐9, while the level of TIMP‐1 is significantly reduced compared to the NC group. However, improvement can be observed through the application of SBP. Experimental results indicate that SBP effectively reduces the expression levels of MMP‐1, MMP‐3, and MMP‐9 in the aging mice's skin while simultaneously increasing the level of TIMP‐1. This action helps restore the balance of the skin and slows down the degradation rate of Col I and tropoelastin. Therefore, SBP may have the potential as an anti‐aging strategy.

### 3.10 Scanning electron microscopic observation of fibrous tissue in the dermis of mice skin tissue

From Figure 5d, we can observe that the fibrous tissues in the dermis of the blank group were tightly connected and structurally ordered. In the model group, the fibrous tissues were looser and more broken, while the fibrous tissues in the SBP‐H group were less broken and more tightly structured than those in the model group. This indicates that SBP can reduce fibrous tissue breakage in the dermis, thus delaying aging.

## 4 DISCUSSION

The stimulation of miscellaneous external factors, such as staying up late, drinking, ultraviolet irradiation, smoking, etc., causes a large accumulation of ROS in cells beyond the normal range, and oxidative stress reactions occur and result in skin problems such as wrinkles, irregular pigmentation formation, and accelerated skin aging (Fitsiou et al., 2021). Therefore, preventing and delaying skin aging has become one of the hot spots and focuses of medical research. More and more studies have focused on the external supplementation of purely natural antioxidants to effectively resist oxidative stress damage and achieve the purpose of delaying aging. From the previous study by our group, we can find that SBP, an oligomeric proanthocyanidin with good free radical scavenging ability, acts as an all‐natural antioxidant and exerts anti‐aging effects by increasing the relative content of Col I in H_2_O_2_‐induced senescence human skin fibroblasts (Liu et al., 2022; Zhu et al., 2021). In this study, we used these findings as the basis to start from the most basic aspects of delaying skin aging, such as the total antioxidant capacity of the skin, the thickness of the skin, the water content of the skin dermis, the content of HA, collagen, and elastin, and the structure of collagen fibers and elastic fibers. Furthermore, the anti‐aging effect of SBP was compared with Vc and GSPE, which are common antioxidants on the market, to study whether SBP has the effect of delaying skin aging from many aspects and elucidate its possible mechanism of action. Further research will help to reveal more potential applications of proanthocyanidins and promote the application of sea buckthorn and its extracts in food, pharmaceutical, and skin care products.

Much experimental evidence shows that aging is related to the body's antioxidant defense capacity decline (Salmon et al., 2010). Proanthocyanidins have a strong free radical scavenging ability to neutralize ROS quickly and reduce damage to cells and tissues from oxidative stress due to high ROS levels (Huang et al., 2022). T‐AOC is the total capacity of the enzymatic antioxidant system and the non‐enzymatic antioxidant system, which is an index to evaluate the overall antioxidant capacity of the body and also an important index to measure the anti‐aging of the body (Zhao et al., 2018). SOD is an important antioxidant enzyme for scavenging ROS in cells, which can catalyze the reduction reaction of the superoxide anion and convert it into more stable oxygen and hydrogen peroxide (Forman & Zhang, 2021). MDA is a product of lipid peroxidation damage in cell membranes and an indicator of the oxidative stress response (Park et al., 2021). The saturated fatty acids in cells are affected by excessive ROS, and an oxidative reaction occurs. MDA is one of the oxidation products of the above reaction, and the concentration of MDA gradually increases with the oxidation reaction (Ito et al., 2019). In our study, we found that injection of D‐galactose in mice increased MDA content in serum and skin and decreased SOD and T‐AOC content, resulting in a decrease in antioxidant defense capacity in vivo, while SBP treatment reversed the above results. In conclusion, SBP can eliminate the excess ROS produced by the oxidative stress response induced by D‐galactose in vivo by improving the total antioxidant capacity of the body, thus achieving the anti‐aging effect.

Whether the skin can remain smooth, refined, and elastic mainly depends on the water content, the content of collagen and elastin in the dermis, and the stability of collagen fibers and elastic fibers. The skin's water content plays a vital role in maintaining the skin's normal function, and the content of the HA in the superficial skin is bound up with the water content. HA initially exists in the skin, which can help the skin absorb water from the body and the skin surface and also enhance the skin's long‐term water retention ability. When the HA absorbs water, it makes collagen fibers and elastic fibers in a moist environment so that the skin has elasticity (Šínová et al., 2022). Collagen is a triple‐helix protein in the ECM and the cell–ECM interface. It is in charge of maintaining the body's structural integrity and the skin's elasticity and tightness. The most abundant collagen is type I, which accounts for 70%–80% and exists in fibrils (Hwang et al., 2021). When Type I procollagen is secreted into the ECM, it undergoes enzymatic cleavage to remove the C‐peptide and N‐peptide, resulting in the formation of mature collagen. These mature collagen molecules have the ability to self‐assemble into highly organized structures known as collagen fibers, providing strength and stability to the skin (Darvish, 2022). On the other hand, elastin, although present in a relatively low amount (about 2% of total skin proteins) (Antonicelli et al., 2007), plays a significant role in skin elasticity. The formation and maturation of elastic fibers occur through a complex process known as “elastogenesis.” During this process, the elastin precursor is deposited and cross‐linked onto a microfibrillar scaffold rich in fibrillin. It is accompanied by a variety of different interactions, such as between proteins or between proteins and other molecules, which can further regulate and stabilize the structure and function of elastic fibers (Schmelzer & Duca, 2022). Therefore, the secretion of collagen and elastin, maturity, and interaction are the keys to maintaining the skin's structure, strength, and elasticity. Understanding these processes in depth is of great significance for the development and application of anti‐aging products and therapeutic approaches that promote skin health.

**FIGURE 6:**
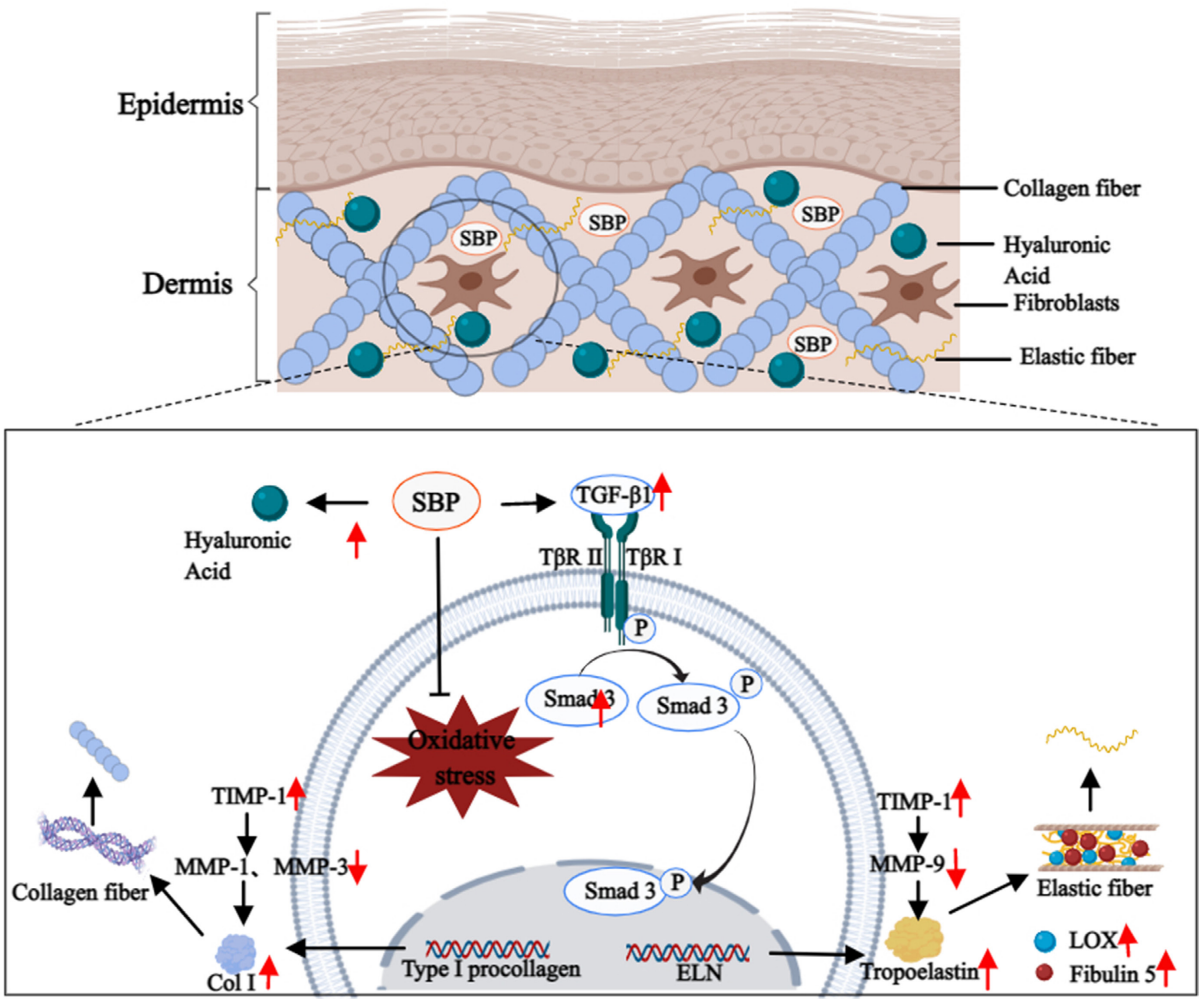
Possible mechanisms of the anti‐aging effect of SBP on the skin. This figure was created with MedPeer.

The relative content of Col I and tropoelastin is essential for maintaining the structural stability of collagen fibers and elastic fibers. The TGF‐β1/Smads pathway is a sensitive pathway for tropoelastin and collagen synthesis. When TGF‐β1 binds to receptors, Smad3 is phosphorylated. Phosphorylated Smad3 is transferred into the nucleus to facilitate Col I and tropoelastin and further promote the synthesis of collagen fibers and elastic fibers (Ignotz & Massagué, 1986; Sárdy, 2009). On the other hand, the MMPs/TIMPs system is essential for regulating the degradation of Col I and tropoelastin (Cavinato & Jansen‐Dürr, 2017; Sproul & Argraves, 2013). Matrix metalloproteinases (MMPs) belong to the zinc‐dependent endopeptidase family, and their activities are affected by TIMPs (Page‐McCaw et al., 2007). It has been shown that the degradation of Col I is carried out by MMP‐1 and MMP‐3 in succession (Cui et al., 2017). While tropoelastin degradation is mainly mediated by MMP‐9 (Yan et al., 2023), TIMP‐1 can inhibit the expression of the above three MMPs (Andraska et al., 2022). Our study focused on exploring the effects of SBP on Col I and the tropoelastin synthesis pathway, as well as the influence of the degradation process. The results showed that in terms of Col I and tropoelastin synthesis, SBP increased TGF‐β1 content in the skin tissue, which in turn upregulated Smad3 expression and promoted Col I and tropoelastin synthesis. In addition, SBP increased the contents of LOX and Fibulin 5, which are essential for synthesizing elastic fibers. Secondly, in terms of Col I and tropoelastin degradation, SBP inhibited the degradation of Col I and tropoelastin in aging skin tissue by regulating TIMP‐1 content in skin tissue cells and alleviating the increase in MMP‐1, MMP‐3, and MMP‐9 content. All in all, SBP can play a positive role in delaying skin aging through two aspects: upregulating the synthesis of Col I and tropoelastin, and inhibiting the degradation of Col I and tropoelastin, thereby effectively improving the aging condition of the skin (Figure 6).

## 5 CONCLUSIONS

From what has been discussed above, in multiple aspects, we evaluated the effective anti‐aging activity of SBP isolated from sea buckthorn and its potential protective mechanism for in vivo senescence induced by D‐galactose. SBP could alleviate the weight loss, skin thinning, and aging score reduction caused by D‐galactose‐induced aging, and it could also strengthen the total antioxidant capacity in vivo by enhancing the activities of antioxidant enzymes. In addition, SBP can upregulate the synthesis of Col I and tropoelastin through the TGF‐β1/Smads pathway and decrease the degradation of Col I and tropoelastin by regulating the MMP/TIMP system. Thus, it can increase Col I and tropoelastin, maintain the stability of collagen fiber and elastic fiber structure, and effectively improve skin aging. We also identified concentration‐dependent effects on the anti‐aging properties of the skin. The results provide new possibilities for developing and producing cosmetics and antioxidant‐functional food products rich in antioxidant functionality.

## AUTHOR CONTRIBUTIONS

**Xinying Liu:** Data curation (lead); methodology (lead); software (lead); validation (lead); visualization (lead); writing – original draft (lead); writing – review and editing (lead). **Michael Yuen:** Funding acquisition (lead); investigation (lead); resources (equal). **Tina Yuen:** Formal analysis (lead); resources (lead). **Hywel Yuen:** Investigation (lead); resources (equal). **Min Wang:** Supervision (lead). **Qiang Peng:** Conceptualization (lead); data curation (supporting); project administration (lead); resources (equal); supervision (equal).

## FUNDING INFORMATION

This research was financially supported by Beijing Technology & Business University (BTBU) and Yulin City Science and Technology Plan Project (No.CXY‐2020‐074) and the Science and Technology Project of Xining (No. 2021‐Y‐15).

## CONFLICT OF INTEREST STATEMENT

Authors M.Y., T.Y., and H.Y. were employed by Puredia Limited. All authors declare no other competing interests.

## ETHICS STATEMENT

All animal protocols used in this study were reviewed and approved by the Animal Policy and Welfare Committee of Northwest A&F University (approval no. XN2023‐0603). The experimental procedures were conducted in accordance with all relevant ethical guidelines for animal experimentation.

## Data Availability

The datasets and materials used in the current study are available from the corresponding author on reasonable request.
